# Gene, Environment and Methylation (GEM): a tool suite to efficiently navigate large scale epigenome wide association studies and integrate genotype and interaction between genotype and environment

**DOI:** 10.1186/s12859-016-1161-z

**Published:** 2016-08-02

**Authors:** Hong Pan, Joanna D. Holbrook, Neerja Karnani, Chee Keong Kwoh

**Affiliations:** 1 Singapore Institute for Clinical Sciences (SICS), Agency for Science Technology and Research (A*STAR), Singapore, 117609 Singapore; 2 School of Computer Science and Engineering, Nanyang Technological University (NTU), Singapore, 639798 Singapore; 3 Yong Loo Lin School of Medicine, National University of Singapore (NUS), Singapore, 119228 Singapore

**Keywords:** Matrix operation, EWAS, methQTL, GxE

## Abstract

**Background:**

The interplay among genetic, environment and epigenetic variation is not fully understood. Advances in high-throughput genotyping methods, high-density DNA methylation detection and well-characterized sample collections, enable epigenetic association studies at the genomic and population levels (EWAS). The field has extended to interrogate the interaction of environmental and genetic (GxE) influences on epigenetic variation. Also, the detection of methylation quantitative trait loci (methQTLs) and their association with health status has enhanced our knowledge of epigenetic mechanisms in disease trajectory. However analysis of this type of data brings computational challenges and there are few practical solutions to enable large scale studies in standard computational environments.

**Results:**

GEM is a highly efficient R tool suite for performing epigenome wide association studies (EWAS). GEM provides three major functions named GEM_Emodel, GEM_Gmodel and GEM_GxEmodel to study the interplay of Gene, Environment and Methylation (GEM). Within GEM, the pre-existing “Matrix eQTL” package is utilized and extended to study methylation quantitative trait loci (methQTL) and the interaction of genotype and environment (GxE) to determine DNA methylation variation, using matrix based iterative correlation and memory-efficient data analysis. Benchmarking presented here on a publicly available dataset, demonstrated that GEM can facilitate reliable genome-wide methQTL and GxE analysis on a standard laptop computer within minutes.

**Conclusions:**

The GEM package facilitates efficient EWAS study in large cohorts. It is written in R code and can be freely downloaded from Bioconductor at https://www.bioconductor.org/packages/GEM/.

## Background

Understanding DNA methylation biomarkers of environmental exposures and developmental trajectories to disease is highly desirable [1] and their discovery is the aim of many epigenome wide association studies (EWAS) [2, 3]. The computational burden in analyzing the genomics data from this type of studies is considerable due to the high number of variables returned from epigenetic screens, for instance >483,000 individual measures from the widely used Illumina Infinium HumanMethylation450 Array (Infinium450K) [4] or the millions of loci covered by RRBS [5] or methyl-capture technologies [6, 7]. Hundreds or thousands of subjects are required to provide the statistical power to draw inference in EWAS studies [8]. The need to include covariates pertaining to the subjects, such as gender, ethnicity and social economic status [9], and to the samples, such as cellular heterogeneity [10–12], increase the computational time needed to run statistical models. Some of these problems are familiar from the genome wide association studies (GWAS) field, although DNA methylation profile is surrogated by continuous percentage values and distributed very differently from genotype calls.

However, what has really pushed EWAS studies to the brink of what is computationally possible, is the realization that DNA methylation levels are not just specified by extrinsic factors but also are influenced by genotype. Polymorphisms close to CpGs in the same chromosome (*cis-)* often form methylation quantitative trait loci (methQTLs) with nearby CpGs [13–15], or blocks of *cis-* polymorphisms associated with a cluster of methylation quantitative trait loci, named GeMES (15, 19). MethQTLs can be discovered by correlating single nucleotide polymorphism (SNP) data with CpG methylation from the same samples. Creating a genome wide methQTL map requires assessing the correlation of genotype at millions of SNPs with thousands to millions of CpG methylation states, by millions multiplied with millions linear iterative regressions. Sun 2014 [16] surveyed methQTL studies between year 2010–2014 and found that most of methQTL studies were restricted to screen *cis-* SNP-CpG pairs, while some were even restricted to the 50,000 bp to 1,000,000 bp regions flanking to each SNP. However SNPs far from the CpG or in different chromosome (*trans-*) were also reported to be associated with CpG. *Trans-* methQTLs have been detected to be relevant to normal or disease states in many studies [17].

Furthermore, it is now apparent that genotype can work in interaction with environment (GxE) to influence specific DNA methylation levels [18, 19] and these can be linked to phenotypes [20, 21]. This type of correlated methylation structure has implications for statistical models whereby genotype and environment, or genotype and methylation interact to predict methylation levels or phenotype. This has exponentially increased the computational burden for the proper analysis of EWAS data.

Large-scale genomic research benefits from high-performance computing (HPC) environments together with parallel computing techniques. However, the operation and integration of results needs domain expertise [22] and HPC is not always easily accessed by biology lab researchers. Therefore, we were motivated to develop computational solutions that allow biological researchers to explore EWAS, methQTLs and GxE using standard desktop computers within realistic computational times.

A R package called MatrixEQTL [23] was developed for expression quantitative train loci (eQTL) analysis. Based on matrix operation, iterative correlation was implemented to achieve computational efficiency, and data was sliced into blocks to achieve memory efficiency. A function in MatrixEQTL that allows inclusion of interaction terms in correlative statistical models, gained our attention, though the author did not highlight it when the package was reported. We deployed the fast and efficient MatrixEQTL software and created a tool suite to explore the associations of Gene, Environment and Methylation. We named the tool suite “GEM”. It provides three fast linear regression models denoted Emodel, Gmodel and GxEmodel to facilitate analyses in EWAS. The GEM_Emodel tests the association of methylome marks and environmental factors; the GEM_Gmodel creates a methQTL genome-wide map; finally, the GEM_GxE model tests the ability of gene and environmental interaction models to predict DNA methylation levels. We benchmarked the performance of the GEM operations on a publicly available EWAS dataset generated on the Infinium450K array with concurrent genotyping on the OmniExpress Array and simulated environment and phenotype information on 237 neonates. Our results demonstrated that the GEM package can facilitate reliable EWAS analyses within minutes, in a standard computational setting (processor = 2.2GHz, RAM = 8G, system = window7 64bit).

## GEM implementation

Simplifying the data input into a methylation matrix as ***M***, genetic variants matrix as ***G***, and the environment vector as ***E***, and the matrix for covariates as ***cvrt***, and using a pseudo coding language like R script, we can denote Emodel (detecting methylation markers associated with environment) function as ***lm (M ~ E + cvrt)***, Gmodel (detecting methylation markers associated with genotype i.e. methQTLs) as ***lm (M ~ G + cvrt)*** and GxE model (interaction of genotype and environment to specify methylation marks) as ***lm (M ~ G×E + cvrt).*** The genome wide studies for Emodel, Gmodel and GxEmodel can be accomplished by calling R function ***lm*** iteratively by millions of times, which were denoted as LM_Emodel (Table 1), LM_Gmodel (Table 2) and LM_GxEmodel.

**Table 1:**
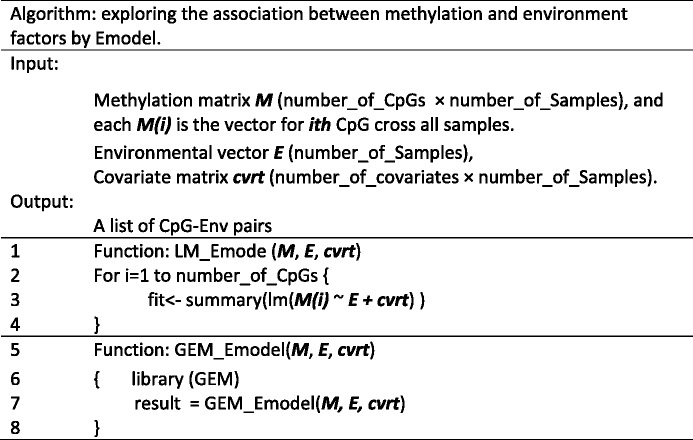
Pseudo R code for Emodel

**Table 2:**
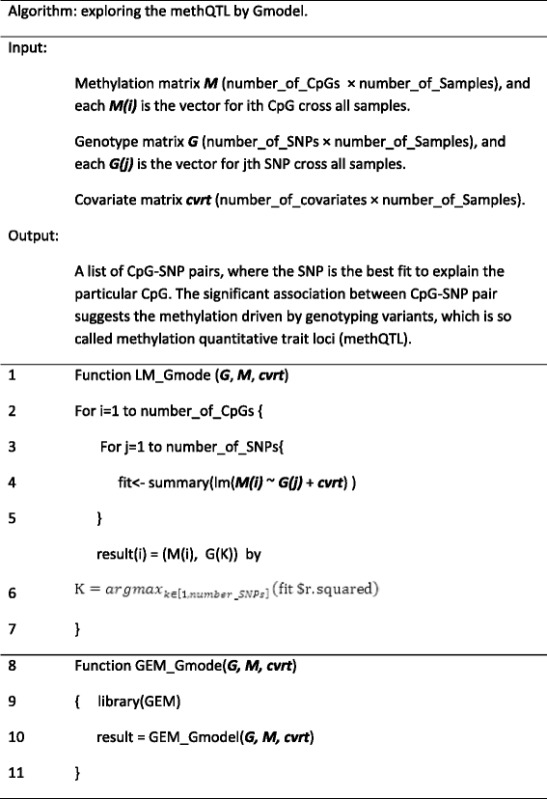
Pseudo R script to explore methQTLs by Gmodel

Shabalin [23] introduced matrix standardization and projection and successfully made an ultra-fast software for expression quantitative trait loci (eQTL). Basically, to quantify the strength of the relationship between ***x*** and ***y*** controlled by covariates (***cvrt***), a practical regression is,

$$ \boldsymbol{y} = \boldsymbol{\alpha} + \boldsymbol{\beta} \boldsymbol{x} + \boldsymbol{\gamma}\ \boldsymbol{cvrt} + \boldsymbol{\varepsilon}, $$

where ***α***, ***β***, ***γ ***and ***ε*** are coefficients, ***β*** is to estimated. A standardization method (22) was applied to vector ***x, y, cvrt***, then the projections of ***x*** and ***y*** to ***cvrt*** are,

$$ \tilde{\boldsymbol{y}}=\boldsymbol{y} - <y,\kern0.75em  cvrt>\kern0.75em  cvrt,\kern0.5em \mathrm{and}\kern0.75em \tilde{\boldsymbol{x}}=\boldsymbol{x} - <x,\kern0.75em  cvrt>\kern0.75em  cvrt, $$

where ***< >*** denotes inner product of two matrix. After these operations, the linear regression between ***x*** and ***y*** with covariates ***cvrt*** can be simplified into the calculation of inner product of the projects of x and y as ${\boldsymbol{r}}_{\tilde{\boldsymbol{x}}\tilde{\boldsymbol{y}}} = <\tilde{\boldsymbol{x}},\ \tilde{\boldsymbol{y}}>$ and estimation of the test statistics.

Shabalin [23] also demonstrated the strategy to slice the large matrix into a small “blocks” in the correlation calculation for memory efficiency, which make the software able to handle data matrix with millions of rows and columns feasible in normal computational setting.

GEM tools called MatrixEQTL [23] library and implemented the below models which were used in [18],

1 $$ \mathrm{GEM}\_\mathrm{Emodel}\ :\boldsymbol{M} = \alpha +\upbeta\ \boldsymbol{E}+\gamma\ \boldsymbol{cvrt}+\upvarepsilon, $$

which was implemented by calling matrixEQTL with “modelLINEAR”, replacing gene expression with methylation, and SNP with environmental data.

2 $$ \mathrm{GEM}\_\mathrm{Gmodel}\ :\boldsymbol{M} = \alpha +\upbeta\ \boldsymbol{G}+\gamma\ \boldsymbol{cvrt}+\upvarepsilon, $$

which was implemented by calling matrixEQTL with “modelLINEAR”, replacing gene expression with methylation.

3 $$ \mathrm{GEM}\_\mathrm{G}\mathrm{x}\mathrm{E}\mathrm{model}:\boldsymbol{M}=\alpha +\upbeta \boldsymbol{G}\times \boldsymbol{E}+\gamma \boldsymbol{cvrt}+\upvarepsilon, $$

which was implemented by calling matrixEQTL with “modelLINEAR_CROSS”, replacing gene expression with methylation.

Emodel finds the association between methylation and environment genome-wide by performing millions of linear regression (N = number_of_CpGs). The output of Emodel for particular phenotype, environmental factor or disease trait is a list of CpGs that are potential epigenetic biomarkers, as in Table 1.

Table 2 demonstrates the pseudo code that used ***lm*** function by iterative loops for Gmodel, we denoted it as LM_Gmodel. The best fit is chosen by the largest R squared value.

Replacing the linear regression equation (line 6) in Table 2 by “fit < − summary(lm(M(i) ~ G(j) * E + cvrt))”, produces the pseudo code for the implementation of LM_GxE model. The output of GxEmodel is a list of CpG-SNP-Env triplets, indicating the CpG-Env association segregated by genotype. The significant association of each triplet implies the methylation change is determined by the interplay between genotyping and environment. Both implementations indicate the number of linear regression as N = number_of_SNPs x number_of_CpGs. N could be billions of linear regressions engendering a very substantial computational task. However, using GEM tools, calculation of methQTLs and GxE interactions can be accomplished with much improved computational efficiency.

## Results

To benchmark GEM suite, we used the dataset from Teh et al. [18]. The standard laptop used for time comparisons had a 2.2GHz processor, 8G RAM, a windows 7 operating system and was 64 bit, which is typical in an academic setting. The HPC structure had eight parallel processes of each with eight core CPUs.

In [18], we studied the 1423 variably methylated regions from the methylomes of 237 neonates, and their association with 708,365 genetic variants and nineteen environmental factors made up of maternal conditions and birth outcomes. The methylome and genotype data are publically available at the NCBI Gene Expression Omnibus (GEO: http://www.ncbi.nlm.nih.gov/geo/ under accession numbers GSE53816 and GSE54445.) Environmental factors were simulated.

**Fig. 1:**
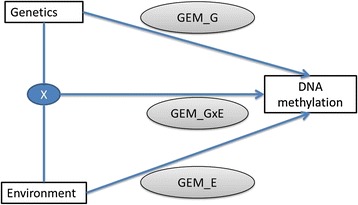
A schematic of the analyses performed within Teh et al. [17]. Gmodel sequentially tests the association of 1423 methylation marks with 708,365 genotypes (and covariates), Emodel sequentially tests the association of 1423 methylation marks with 19 environmental factors and GxEmodel sequentially tests that association of 1423 methylation marks with the interaction of every combination of the 708,365 genotypes and 19 environmental factors.

A schematic of the analyses performed is shown in Fig. 1. When the original analyses were conducted, multivariate regression models were applied sequentially in a HPC environment. For the same dataset, we compared the time taken to implement LM_Gmodel, LM_GxEmodel and LM_Emodel in a standard and HPC computational environment with the time taken to implement GEM_Gmodel, GEM_GxEmodel and GEM_Emodel on a standard laptop (Table 3).

**Table 3:** Benchmarking time consumption of GEM implementations on Emodel, Gmodel and GxEmodel by comparing normal R script in a public available dataset in standard laptop and HPC settings

	Dataset: Teh et al., 1423 CpGs, 708,365 SNPs and 19 environments in a standard laptop			
Method	Time cost on standard laptop	Time cost in HPC	Method	Time cost
LM_Emodel	95.1 s		GEM_Emodel	18.9 s
LM_Gmodel	> = 60 days (^a^)	3 h	GEM_Gmodel	5.2 min
LM_GxEmodel	> = 60 days (^a^)	21 h	GEM_GxEmodel	1.5 h

^a^The time for LM_Gmodel and LM_GxEmodel in standard laptop was computed based on the time cost on 10 CpGs

### Benchmarking GEM_Emodel

**Fig. 2:**
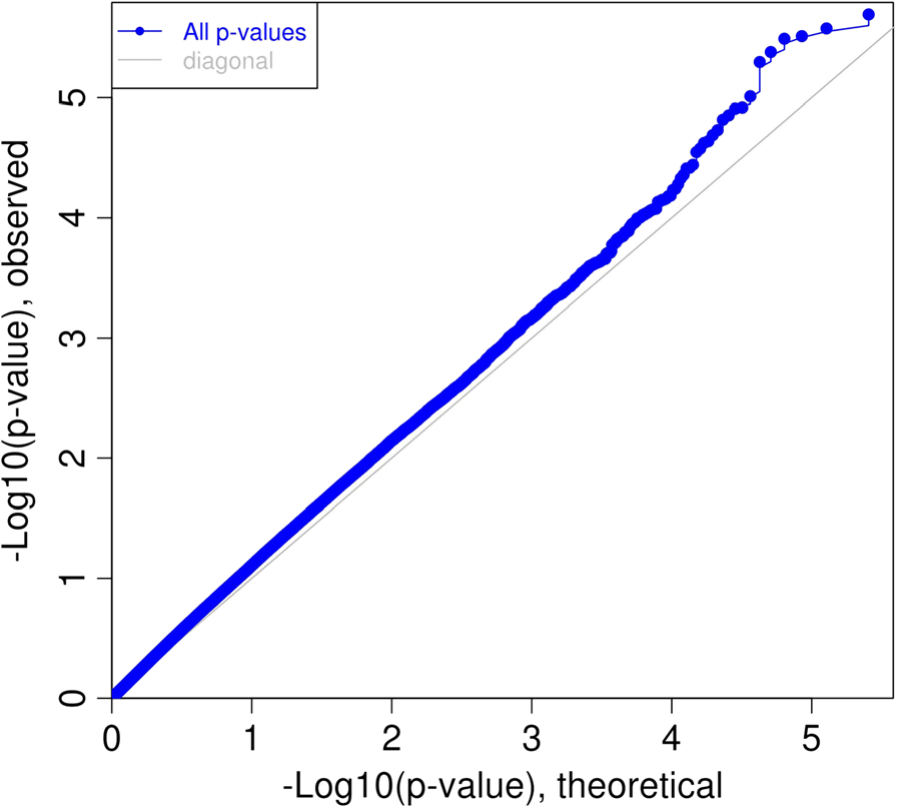
QQplot for pvalues from iterations of ~250,000 CpGs with an environment factor. It was produced by GEM_Emodel

A substantial time saving was achieved using GEM_Emodel compared to standard sequential regression as LM_Emodel as in Table 1. (19 s compared to 95 s for 19 Emodels on 1423 CpGs). Results achieved were identical. In addition, GEM_Emodel has the option to create Q-Q plot for theoretical distribution and observed distribution on p values for every environment e.g. Fig. 2.

**Fig. 3:**
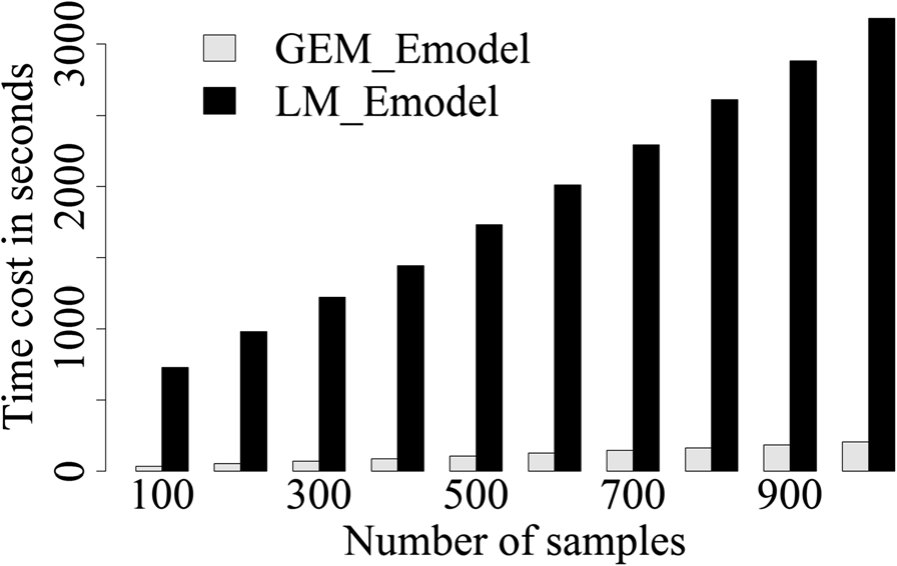
The operation time consumption benchmark on the associations for ~250,000 CpGs and one environmental factor and one covariate for the sequential number of samples from 100 to 1000. X-axis is the number of subjects, and y-axis is the time consumption in seconds. The benchmarking is done in a personal computer (processor = 2.2GHz, RAM = 8G, system = window7 64bit)

As subject numbers increase, computational time to run sequential models increases exponentially, whilst computational time in GEM_Emodel increases linearly. Figure 3 shows the computational time required for one Emodel on 100–1000 subjects for ~250,000 CpGs.

### Benchmarking GEM_Gmodel

In the original analysis [18], the regression equation (Eq. 1 and Table 1) used built-in lm function in R script, which we denoted as LM_Gmodel, was applied to each of the 1423 VMRs, cycling through the 708,365 SNPs, adjusted by sex as the covariate, resulting in 1008 million regression models. We compared the LM_Gmodel with GEM_Gmodel by the result and computational efficiency in a standard laptop (processor = 2.2GHz, RAM = 8G, system = window7, 64bit). We also used a HPC structure with eight parallel processes of each with eight core CPUs (denoted as HPC) to benchmark LM_Gmodel as a reference. The computational time on HPC was 3 h, in a standard computational environment, computational time was estimated to be 61 days. The same data was processed by the GEM Gmodel. It took 5.2 min to accomplish the task on a standard laptop. The results were identical to those reported by Teh et al. [18] i.e. 12 disrupting pairs, 828 in *cis-* pairs and 583 in *trans-* pairs.

### Benchmarking GEM_GxEmodel

The same scale of improvement in performance was achieved for the GEM_GxEmodel where each CpG was tested against the interaction of genotype at each of 708,365 SNPs with each of 19 environmental factors. This analysis originally took 21 h in the HPC environment and an estimated > =60 days on a standard laptop by using normal linear regression in R script, denoted as LM_GxEmodel. In the GEM_GxEmodel, it was accomplished in only 1.5 h. The results were identical between analyses with identical p-values for models containing all winning pairs of SNPs and environments (data not shown).

**Fig. 4:**
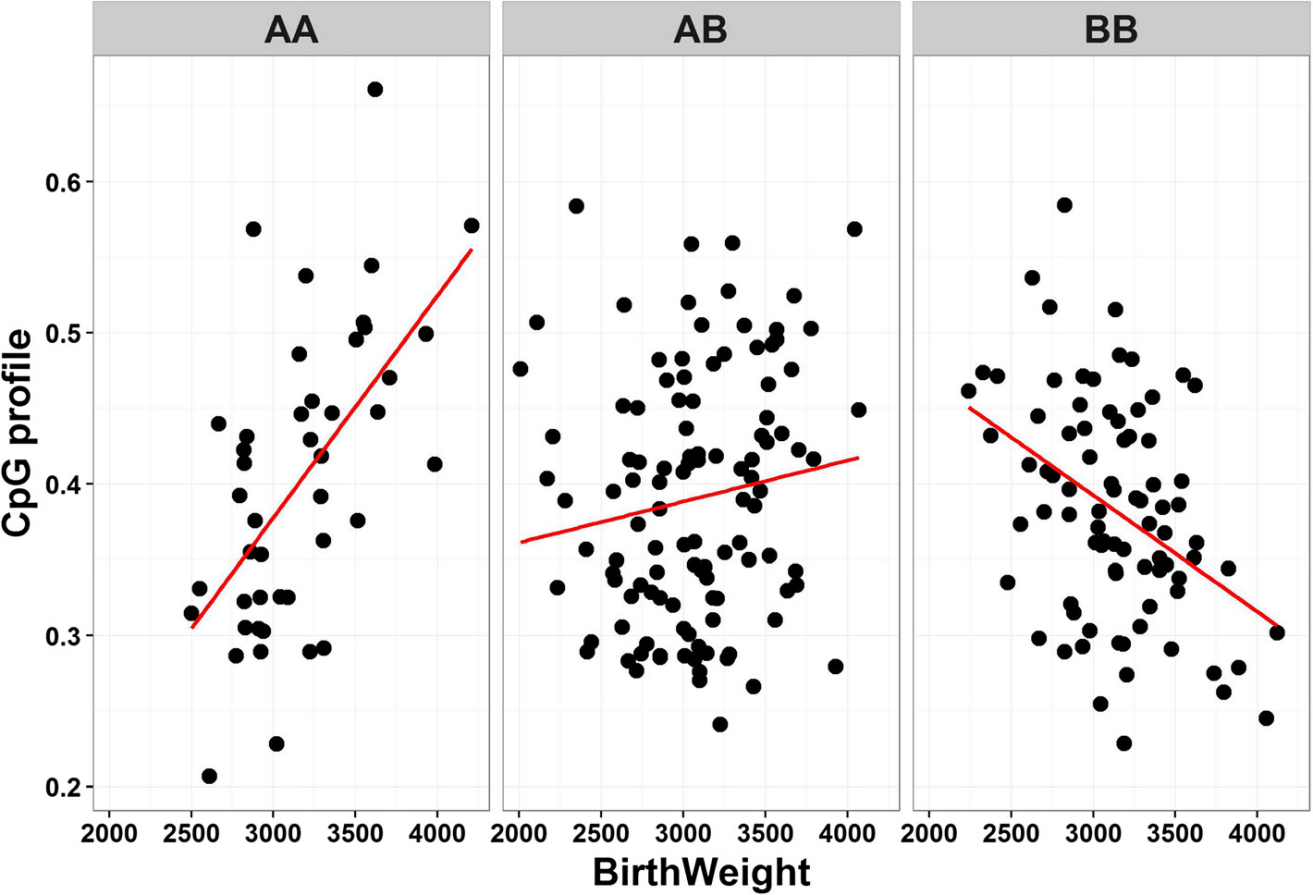
The scatter plot to display an example of methylation corresponding to the environment in different genotype groups. AA, AB and BB are pseudo codes for major allele homozygote, heterozygote and minor allele homozygote. Phenotypic values are shown on the x-axis, and methylation value in percentage on the y-axis. The straight lines fit for associations in each group

In addition GEM also has the option to produce a “segregation scatter plot” for methylation corresponding to environment in different genotype groups, for example, Fig. 4.

## Conclusion and discussion

The advancements in genome-wide genotyping and DNA methylation assessment methods, coupled with well-characterized biological samples enable epigenetic association studies. GEM is designed for very fast testing of millions of hypotheses in epigenetics by using multiple linear regression models. It is suitable to the standard computing resources available to nearly all researchers.

GEM includes a graphic user interface for the convenience of researchers and does not require specialist computational knowledge, outside of the widely used R environment.

**Fig. 5:**
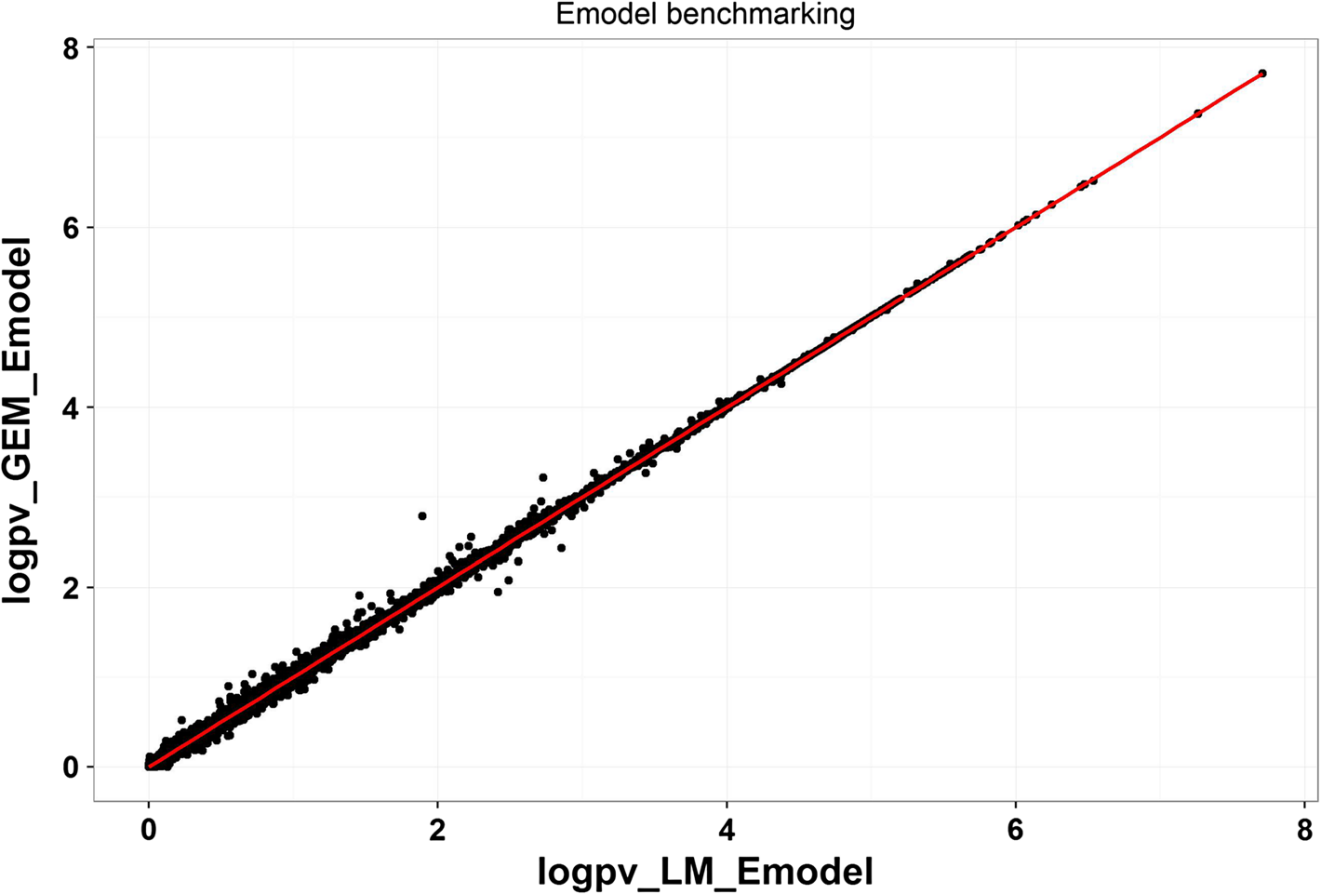
Emodel benchmarking for methylation matrix containing missing values. Pvalue was transformed as –*log*10. A-axis is pvalues from LM_Emodel, y-axis is from GEM_Emodel. Among ~250,000 CpGs that were tested, 18 % of them contained at least one missing values. Our results showed pvalues for CpGs without missing values are perfectly matched, while there were slightly differences between the two implementations when CpG contains missing values

It should be noted that missing data requires careful handling in matrix-based operations. GEM uses the mean value to impute missing values if the data matrices supplied are incomplete. Figure 5 showed the p-values for GEM_Emodel and LM_Emodel are slightly different when the methylation matrix contains missing values. Researchers should assess the suitability of this imputation in the context of the individual study.

## Abbreviations

*cis-*, SNP and CpG locate in the same chromosome; EWAS, Epigenome wide association studies; GxE, the interaction of environmental and genetic influences; methQTLs, methylation quantitative trait loci; *trans-*, SNP and CpG locate in different chromosomes
